# Building a better model of cancer

**DOI:** 10.1186/1747-1028-1-24

**Published:** 2006-10-18

**Authors:** Andriy Marusyk, James DeGregori

**Affiliations:** 1 Department of Biochemistry and Molecular Genetics, Department of Pediatrics, Integrated, Department of Immunology, Program in Molecular Biology, University of Colorado at Denver and Health Sciences Center, Aurora, CO 80045, USA

## Abstract

The 2006 Cold Spring Harbor Laboratory meeting on the Mechanisms and Models of Cancer was held August 16–20. The meeting featured several hundred presentations of many short talks (mostly selected from the abstracts) and posters, with the airing of a number of exciting new discoveries. We will focus this meeting review on models of cancer (primarily mouse models), highlighting recent advances in new mouse models that better recapitulate sporadic tumorigenesis, demonstrations of tumor addiction to tumor suppressor inactivation, new insight into senescence as a tumor barrier, improved understanding of the evolutionary paths of cancer development, and environmental/immunological influences on cancer.

## Background

### New mouse models: keeping it real

Traditional mouse models of cancers initiated by oncogene activation or tumor suppressor inactivation have yielded valuable insight into tumorigenesis, but have suffered from concerns that the mutation in either all cells of the mouse or in all cells of a particular tissue fails to properly recapitulate natural cancer initiation. In the last few years, new models have been developed that rely on Cre recombinase mediated deletion of floxed sequences to activate an oncogene or inactivate a tumor suppressor in only a subset of cells of a tissue at a defined point in time. Published studies have used Cre mediated activation of mutant K-Ras (by removal of STOP sequences), with or without similar mutation in p53, to generate models of lung adenocarcinoma in mice [1-3]. These and similar models are more relevant to spontaneous tumorigenesis, as cells bearing the mutation are surrounded by non-oncogenically mutated competitors, and there are reduced concerns for developmental compensation effects associated with standard knockout or transgenic models.

Analogous to the Ras model, **D. Dankort (A. McMahon lab) **showed that activation of oncogenic BRAF^V600E ^via adenoviral delivery of the Cre recombinase in the mouse lung leads to frequent neoplastic lesions, albeit benign with proliferation indexes that decrease with time, suggesting that additional oncogenic hits are required for progression to full malignancy. **A. Puzio (C. Abate-Shen lab) **developed a mouse model for bladder carcinoma, histologically similar to the human disease, using adenoviral delivery of Cre to induce simultaneous p53 and PTEN deletion in the bladder *in vivo*. As will be discussed in the next section, conditional models for gene inactivation can also be used to determine the importance of maintaining tumor suppressor inactivation in an established tumor.

While stem cells are thought to represent the targets for tumorigenesis for at least some cancers, more committed progenitors may also be targets, particularly if early mutations confer self-renewal. **R. Wechsler-Reya **showed that conditional deletion of Patched (resulting in activation of the Hedgehog pathway) in lineage-restricted granule neuron precursors (GNPs), while not sufficient for transformation of all cells, does lead to medulloblastomas in all mice. Thus, these cancers can arise in more lineage-restricted neuronal progenitors, although further studies will be needed to determine whether they actually do in humans. Unlike the Lox-Cre models discussed above, Patched should be deleted in most GNPs, and since GNPs without Patched are continuously replenished, there is ample opportunity for additional mutations that result in transformation.

## Exploiting the tumor addiction to p53 inactivation

Genetic analysis of cancers reveals a number of defined mutational events that appear specific for particular cancers, indicating that cancers follow certain evolutionary paths. However, the fact that a particular mutation is selected at a certain step in cancer development does not necessarily imply that the same mutation is required for tumor maintenance. The question of whether a particular mutation is important for maintaining a full-blown malignancy is important not only for understanding the biology of cancer, but also for devising rational treatment strategies. For several oncogenes the requirement for maintenance of an initiating mutation for cancer viability has been experimentally demonstrated, leading to the term "oncogene addiction" [4], and thus making these mutants attractive therapeutic targets. However, a similar dependency has thus far not been documented for tumor suppressor genes. Addressing this deficiency, several presentations at this meeting demonstrated that tumor cells must maintain mutant p53 to survive and proliferate.

**C. Martins **(**G. Evan **lab)explored the importance of loss of p53 function for leukemia maintenance using tamoxifen (TAM)-inducible p53 (p53ER^TAM^) [5] in spontaneous leukemias arising in Eμ-Myc transgenic mice in a p53ER/+ background. These Eμ-Myc induced lymphomas typically disable the p53 pathway. Thus, tumors arise in an initially functional p53 background, with p53 disruption selected for during lymphoma progression. Restoration of p53 function by TAM (allows apparently normal p53 activity to be produced from the p53 ER allele) induces rapid apoptosis in leukemic cells, but eventually leads to selection of escapees that are insensitive to p53 restoration. Combining p53 restoration with extrinsic p53 activation stimuli (γ-irradiation) leads to substantial improvement in inhibition of cancer progression.

**A. Ventura (T. Jacks **lab) employed a knock-in mouse experimental system, where endogenous p53 expression was prevented by Lox-flanked transcriptional termination sequences, in order to address the consequence of re-expressing p53 in spontaneous or irradiation induced malignancies that arise on a p53 null background. In this system, endogenous p53 expression can be restored by action of Cre recombinase conditionally activated by tamoxifen expression. Restoration of p53 expression led to dramatic inhibition of tumor progression and regression of the tumors. Interestingly, the effect of p53 restoration appeared to be tissue specific: p53 re-expression in lymphomas induced rapid apoptosis, while in carcinomas it induced permanent senescent-like arrest. In both cases, restoration of p53 function delivered a fatal blow to the tumor.

Two reports from **S. Lowe's **lab reached similar conclusions using conditional expression of an shRNA against p53. **W. Xue **used a liver progenitor cell transplant model developed by **L. Zender **in the Lowe lab [6] to explore the dependence of liver carcinoma induced by oncogenic Ras expression in the absence of p53 on the maintenance of p53 deficient status. Restoration of p53 activity (even transient) led to complete regression of aggressive liver tumors. Surprisingly, the carcinoma cells did not undergo apoptosis, but instead displayed features of senescent-like arrest. Although the arrested cells could maintain their viability *in vitro *for a very long time, *in vivo *the tumors regressed very rapidly. Expression profiling revealed that the senescent tumor cells dramatically upregulated expression of pro-inflammatory leukocyte-attracting cytokines and immune receptors. Activation of p53 resulted in infiltration of the tumor mass with immune cells (granulocytes, macrophages and neutrophils), and application of chemical inhibitors of macrophages and neutrophils substantially delayed tumor regression, revealing the importance of the innate immune system in removing senescent cells. Analogous to results from T. Jacks lab, restoring p53 expression by silencing the shRNA silencer in B-cell lymphomas induces apoptotic death of tumor cells (**R. Dickins **in Lowe lab). In summary, results from the Jacks, Lowe and Evan labs using different conditional models all validate p53 as an attractive therapeutic target.

In a related story, **G. Evan **presented evidence that p53 stabilization due to the absence of Mdm2, even without numerous modifications of the p53 protein induced by stress, can rapidly induce senescence or apoptosis in an adult animal. Protein levels of p53 are held in control by its own target MDM2, which both squelches protein activity and down-regulates the protein levels via proteosomal degradation. Null mutation of MDM2 leads to early embryonic lethality unless rescued by p53 mutation [7,8]. Evan used the p53 ER-TAM system to restore p53 expression in the MDM2 knockout background in adulthood. Restoration of p53 expression (even with a single dose of tamoxifen) led to rapid apoptosis of all radiosensitive tissues resulting in the death of the mice within 6 days. Interestingly, radioresistant tissues maintain their viability, even when restoration of p53 expression was combined with DNA damage. Instead of undergoing apoptosis, radioresistant tissues underwent permanent proliferation arrest, again highlighting intrinsic tissue-specificity in p53-dependent cell fates.

It appears that in some cases "oncogene addiction" might be tightly linked with "suppressor inactivation addiction". **L. Beverly **(**A. Capobianco **lab) presented evidence that Notch (which is mutated in 50% of human T-ALL) inactivates p53 via repression of ARF-dependent MDM2 regulation. Inactivation of Notch in spontaneous tumors arising in mice that conditionally express Notch in the T-cell compartment leads to rapid tumor regression via p53 dependent apoptosis. Importantly, p53 can be reactivated using either irradiation or Nutlin even in the presence of sustained Notch signaling, resulting in apoptosis and lymphoma regression. Thus, it is proposed that inhibition of p53 activity by Notch signaling might be an important step in the evolution *and *maintenance of the resulting T cell malignancies. These findings provide a basis for the high responsiveness of tumors with aberrant Notch signaling to agents that re-activate p53, such as Nutlin or irradiation.

While others have shown that persistent expression of Myc or Ras oncogenes is required for tumor maintenance [9], **K. Podsypanina **(**Varmus **lab) demonstrated that in mammary tumors resulting from coexpression of activated K-Ras with either Myc or Wnt-1, deinduction of K-Ras (using tetracycline regulation) leads to rapid tumor regression. Thus, oncogene addiction extends to contexts where multiple defined initiating events are involved in tumorigenesis, which is perhaps not surprising given that oncogene addiction demonstrated in other models is on the background of additional, albeit undefined, oncogenic hits.

Finally, **J. Jonkers **reported on the development of conditional mouse models of breast cancer. By K14-Cre (expressed in epithelium) mediated deletion of p53, together with either E-cadherin or BRCA1, they demonstrated enhanced breast and skin carcinogenesis relative to p53 deletion alone. However, the mechanisms may be distinct, with E-cadherin loss abrogating detachment induced apoptosis (anoikis) and BRCA1 loss resulting in increased genomic instability. As the resulting adenocarcinomas resemble their human counterparts, these models should be useful for testing therapeutics and drug resistance mechanisms. Indeed, in their BRCA1/p53 mammary tumor model, the development of drug resistance appeared to derive from enhanced multidrug resistance pump activity.

## Cellular senescence

From its discovery, cellular senescence has been thought to be an important barrier for cancer development. Recent reports [10-14] have demonstrated the validity of such speculation, establishing oncogene-induced cellular senescence as a physiologically relevant tumor suppression mechanism. Presentations from the T. Jacks, G. Evan and S. Lowe labs reviewed above highlighted that choice of apoptosis vs. senescence is in large part cell-type specific.

While permanent senescent growth arrest has unambiguous effects at the cellular level, it also leads to dramatic changes in gene expression. The classical marker for senescence has been the expression of senescence associated β-galactosidase activity, and more recently senescence has been associated with DNA damage foci [15]. One important aspect of senescence associated gene expression changes is the "secretory phenotype", involving substantial increases in the secretion of biologically active molecules including inflammatory cytokines, metalloproteases and growth factors, which lead to non-cell autonomous effects resulting from cellular senescence [16]. A number of talks addressed the senescent cell secretion phenotype and its biological significance, suggesting that the secretory phenotype could either promote or suppress cancer progression in different contexts.

**Judith Campisi's **lab has previously shown that the secretory phenotype displayed by senescent fibroblasts can substantially promote tumorigenesis by stimulating the proliferation and tumorigenicity of pre-malignant cells [17,18]. Her presentation explored a more molecular characterization of this phenotype. Microarray data indicated that the secretory phenotype is shared by senescent fibroblasts from different tissues and donor ages, as well as senescent endothelial and mammary epithelial cells. However, the intensity of the secretory phenotype did vary with different senescence-causing stimuli. Interestingly, disruption of p53 in primary human fibroblasts that underwent replicative senescence leads to dramatic further up-regulation of the secretory phenotype coinciding with the expected reversal of senescence, revealing a non-cell autonomous tumor suppressor function of p53.

Other reports however, implied anti-tumor functions of the secretion phenotype. As described above, studies by **W. Xue **and **S. Lowe **using a hepatocarcinoma model revealed that secretion of inflammatory cytokines mobilized the innate immune system to eliminate senescent tumor cells, thus perhaps preventing the emergence of mutants escaping the senescence. **D. Peeper's **lab reported an important role of cytokines secreted by senescent cells in maintaining the senescent phenotype. This group recently published that human naevi (benign tumors of melanocytic origin that can remain in unaltered state for decades and often express BRAF^V600E^) display senescence-like growth arrest [11]. In his current talk, Peeper described studies to identify mechanisms responsible for maintaining the irreversibility of arrest as well as events that allow cells to escape the senescent-like arrest triggered by oncogene expression (BRAF^V600E ^expressed in p16 mutant fibroblasts). Gene-expression arrays identified potent induction of interleukins (IL-6, IL-1α, β). Importantly, they showed that expression of IL-6 and its receptor were required for maintenance of the senescent phenotype. C/EBPβ was identified as a common denominator responsible for induction of cytokines and maintenance of senescence. The use of bar-coded shRNA library screen for senescence-escape mutants allowed identification of additional genes implicated in maintenance of senescence, and thus potentially oncogenic events that can allow malignant transformation.

## Tumor evolution

The last few decades have seen dramatic progress in advancing a gene-centered understanding of molecular mechanisms of cancer development, including elucidation of pathways that regulate cellular proliferation, apoptosis and growth arrest. While better understanding of cell-intrinsic mechanisms and pathways is undoubtedly a step toward better clinical strategies, excessive "gene-centrism" initially gave insufficient attention to the fact that the evolution of cancer is shaped not only by cell-intrinsic events, but also to a large extent by the environment. **M. Bissell **overviewed critical roles that the extracellular environment has on the development of cancers, highlighting evidence that alteration of physiological communication of cancer cells with the environment is not only a required step in cancer evolution, but also that the disruption of this communication impacts other hallmarks of cancer, such as proliferation and genetic instability [19]. Importantly, restoration of normal communication at least in some cases can lead to dramatic reversal of cancer phenotypes. Moreover, macroscopic structure and polarity have a substantial impact on cancer biology, evolution and outcomes of therapy. Therefore, understanding the mechanisms that allow communication between macroscopic structures and microscopic events is of great importance. Bissell also presented interesting studies of epithelial branching development using 3-d gels of set microscopic shapes. Macroscopic structure was found to impose strict restraints on local outgrowth of cells. Combination of bioinformatics methods and experimental approaches allowed identification of a TGF-β inhibitory gradient as being the major factor responsible for macroscopic structure regulation of local morphogenesis.

Cancer progression can undoubtedly be regarded from the perspective of principles of Darwinian evolution. In order for an oncogenic mutation to be selected it should increase the fitness of the cell. But the fitness of a cell is determined by not only cell-intrinsic properties but also by the environment where the cell exists and by the relative fitness of other cells in the same population, since selection works by competition. **J. DeGregori**'s lab has previously demonstrated that a background of impaired cellular proliferation engendered by drugs or genetic alterations substantially increases the selective advantage provided by the Bcr-Abl oncogenic translocation, resulting in dramatic increases in leukemogenesis [20]. Aging is the single strongest prognostic factor for cancer predisposition, which has largely been explained by accumulating mutations with age. Still, age is also clearly associated with the decreased function of progenitor cells in many tissues, suggesting the possibility that "poor fitness" of cell populations might be a factor contributing to the dramatic increase in the onset of malignancies observed with advanced age. **A. Marusyk **from the DeGregori lab presented evidence supporting the validity of this hypothesis. Using a bone marrow transplantation model for leukemia initiation, Bcr-Abl was demonstrated to provide a much stronger selective advantage in the background of aged hematopoiesis compared to the background of young hematopoiesis, leading to a substantial increase in leukemias. Importantly, this age-dependence appeared largely non-cell autonomous, since introduction of young competitors efficiently reversed the competitive advantage conferred by Bcr-Abl expression. These findings reveal a novel factor contributing to age-related increase in cancer incidence, highlighting the importance of non-cell autonomous forces driving cancer evolution.

While experiments described above showed that mutations (such as in p53) involved in the genesis of cancer can also be required for tumor maintenance, an interesting presentation from **B. Amati's **lab suggest that tumors could select for the restoration of partial loss of function events that favored tumor initiation. **C. Gorrini **presented evidence that the acetyl-transferase Tip60 is important for Myc-induced double-stranded DNA damage response, as heterozygosity for Tip60 substantially attenuates activation of p53 and ATM. Tip60 heterozygosity accelerates the onset of Eμ-Myc driven lymphomagensis, suggesting the importance of DNA damage response in the suppression of tumorigenesis. Although Tip60 heterozygosity prevents activating phosphorylation of p53, lymphomas originating in a Tip60+/- background still retain selective pressure to lose p53. Interestingly, while Tip60 heterozygosity dramatically shortens the onset of lymphomas, the lymphoma cells duplicate the remaining Tip60 allele, restoring Tip60 expression to wildtype levels. These studies suggest an interesting scenario, whereby haploinsufficiency is important in the initial stages of tumor development, but at some point in cancer evolution there is selective pressure to restore gene function.

Evolution of cancer involves acquisition of multiple new traits, the so called "hallmarks of cancer" [21]. Each hallmark is traditionally though to be associated with new alterations in the cancer cell genome. Using the reversible transgenic model to explore c-Myc driven oncogenesis in pancreatic β-cells, **G. Evan **has previously shown that activation of Myc on the background of suppressed apoptosis not only drives cell proliferation and expansion of β-cells, but also concomitantly induces features of advanced neoplasia such as increased invasiveness and vascularization [22]. Here Evan presented findings revealing the mechanisms of increased angiogenesis. Myc expression led to translocation of VEGF from extra-cellular matrix to vessel epithelia [23]. This re-localization was dependent on Myc-induced increases in production of IL-1β and inflammatory cytokines that recruited mast cells to adjacent stroma. Prevention of migration of mast cells efficiently blocked angiogenesis, leading to hypoxia induction in Myc-expressing β-cells and blockade of islet expansion. These findings highlight the importance of communication within the local environment in c-Myc driven angiogenesis and oncogenesis.

## Reducing immune system tolerance to target cancers

**J. Allison **presented a report of the successful mobilization of the immune system to target cancer cells by capitalizing on a better understanding of the complex regulatory pathways that coordinate T-cell immune responses. The immune system is extremely efficient in eliminating cells that express foreign or abnormal proteins. Extensive research efforts have been applied to harness the immune system to recognize and eliminate cancer cells, and although T-cells are capable of recognizing tumor-specific antigens, attempts to mobilize T-cells to kill tumor cells have thus far met with very limited success. Part of the problem appears to lie in the existence of intricate control mechanisms that negatively regulate T-cell proliferation in order to prevent autoimmune disease. One such control mechanism involves upregulation of CTLA-4 receptor in response to antigen receptor signaling [24]. CTLA-4 upregulation antagonizes T-cell signaling, proliferation and activation. While CTLA-4 protein normally has a very short half-life, chronic antigen presentation leads to maintained CTLA-4 expression and thus continuous inhibition of T-cell response. Tumors may present such chronic stimulation which can lead to host tolerance of the tumor. Allison presented evidence that blocking CTLA-4 signaling can dramatically improve T-cell mediated killing of tumor cells, leading to development of effective clinical strategies. While some tumors have intrinsically poor immunogenicity and avoid immune response even when CTLA-4 is blocked, combining CTLA-4 blockade with vaccination against tumor-specific proteins and/or with therapies that lead to increased death of tumor cells (which also boost presentation of tumor proteins by antigen-presenting cells) can substantially improve the recognition and elimination of such tumors. In fact, durable, objective responses have been obtained in melanoma, renal, prostate, and ovarian cancer in clinical trials.

The results presented above highlight substantial progress in developing and refining experimental models of cancers. The studies reviewed here emphasize the importance of the research shift towards animal modeling in order to understand not what *might possibly *be happening but what *really is *happening (to paraphrase M. Bissel). In addition, the cancer field is showing steady progress towards a better understanding of the evolutionary forces that drive tumorigenesis, the intricate and inherent tumor suppressive mechanisms that prevent cancer, and the alterations that allow tumor cells to escape these controls. Building upon this knowledge will allow a more intelligent identification of targets for anti-cancer therapies and strategies for cancer prevention.

## Competing interests

The author(s) declare that they have no competing interests.

## Authors' contributions

AM and JD both researched and wrote this meeting review.
